# Evaluating the use of SPIRIT on participant retention in randomised trials: Challenges in reporting and implications for practice

**DOI:** 10.1371/journal.pone.0327110

**Published:** 2025-08-12

**Authors:** Amy O’Connor, Ellen Murphy, Frances Shiely

**Affiliations:** 1 Trials Research and Methodologies Unit (TRAMS), Health Research Board Clinical Research Facility, University College Cork, Cork, Ireland; 2 School of Public Health, University College Cork, Cork, Ireland; Faculty of Medicine Vajira Hospital, Navamindradhiraj University, THAILAND

## Abstract

**Objectives:**

To establish whether the use of SPIRIT (Standard Protocol Items: Recommendations for Interventional Trials) item 18b (retention), evidence of planning for retention, has any effect on participant retention rates in randomised controlled trials.

**Study design and setting:**

A retrospective study of randomised controlled trials between 2014 and 2019. We reviewed 506 trial protocols, 253 protocols that stated the use of SPIRIT guidelines in the protocol, and 253 protocols that did not.

**Results:**

The reported use of SPIRIT guideline item 18b in the trial protocol has no significant effect on participant retention rates in the corresponding trials. It does not impact the overall retention rate of participants throughout the trial, nor the retention rate of the primary outcome measure.

**Conclusion:**

SPIRIT item 18b appeared not to contribute to improved participant retention rates in randomised trials. However, the lack of high-certainty evidence for effectiveness of the intervention strategies cited likely impacts this finding. Additionally, inconsistent reporting of retention strategies in trial protocols is evident and needs to be more complete to facilitate evaluation.

## Introduction

Retention is a considerable challenge in randomised controlled trials [1–3]. Retention issues have been identified as one of the top research priorities in trials methodology [4] and require a solution that can be implemented globally. Poor participant retention impacts the quality and validity of the trial results, makes it difficult to draw accurate trial conclusions, causes trial delays and increases trial costs [1,5–8].

We previously conducted a scoping review [9] which highlighted the lack of communication of plans to promote participant retention in trial protocols. In that, we identified inconsistent reporting of SPIRIT (Standard Protocol Items: Recommendations for Interventional Trials) item 18b. SPIRIT is a set of guidelines introduced in 2013 that provide a framework for designing and reporting randomised trials, and item 18b is specifically concerned with participant retention. While SPIRIT is widely endorsed as an international standard for trial protocols [10], no prior research exists on the effectiveness of the completion of SPIRIT item 18b in the protocol on participant retention in the corresponding trial report.

The purpose of this study was to examine if the completion of SPIRIT item 18b, i.e., evidence of planning for retention, in the protocol improves the retention of participants in the subsequent trial. We were also interested in knowing if those that don’t complete SPIRIT in their trial protocol report its use in their trial publication.

## Materials and methods

**Fig 1:**
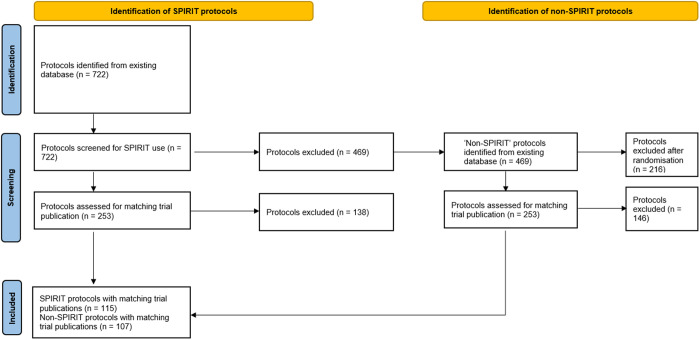
PRISMA flow diagram of protocol and corresponding trial publication selection.

As part of a previous scoping review [9] we collected trial protocols, some of which mentioned the use of the SPIRIT guidelines when developing the protocol and some of which did not. To compare whether the reporting of SPIRIT item 18b improves retention, we selected all the protocols that reported the use of the SPIRIT guidelines and randomly selected a matching group of protocols that did not report the use of SPIRIT guidelines. We searched PubMed and Medline for the corresponding trial results papers, published as a journal article. Where journal articles could not be accessed, the corresponding author of the trial protocol was contacted in an attempt to obtain the publication. In some instances, the trials were still actively recruiting or had not yet been completed, so, these journal articles could not be included. Fig 1 shows the PRISMA flow diagram for the selection of protocols and corresponding trial publications.

### Data extraction

All authors met and agreed the variables to be extracted from the trial publications. This included trial publications that mentioned using the SPIRIT guidelines (hereafter called ‘SPIRIT trials’) and those that did not mention using SPIRIT (hereafter called ‘non-SPIRIT trials). These are shown in Table 1. We defined a retention strategy as per our definition in our scoping review “an action/activity that is conducted with the purpose of retaining participants in a trial, reducing missing data or improving data completeness”(9:3). We were not concerned with extracting information regarding activities to improve adherence or compliance with an intervention.

**Table 1:** Variables extracted from the journal articles.

Variable	Description
Use of SPIRIT?	Did they mention they used SPIRIT to develop their protocol in the published journal article?
Numbers recruited?	N/A
Numbers retained overall?	Numbers retained where PO was not the final time point of assessment (i.e., a secondary or tertiary outcome)?
Numbers retained to the primary outcome?	N/A
Point of consent/point of randomisation?	Did we capture the point of consent or the point of randomization?
CONSORT flow diagram present?	CONSORT flow diagram present and therefore used to obtain data
Is any retention strategy mentioned in the journal article?	If yes, copy and paste details of that retention strategy from the journal article.
Reported item 18b(i)	Plans to promote participant retention.
Reported item 18b(ii)	Plans to complete follow-up including any outcome data to be collected for participants who discontinue from intervention protocols.
Reported item 18b(iii)	Plans to complete follow-up including any outcome data to be collected for participants who deviate from intervention protocols.
Reported all 3 items of SPIRIT 18b	N/A

AOC trialled the data extraction from the trial publication with five ‘SPIRIT trials’ and five ‘non-SPIRIT’ trials. EM double extracted the same trials. Five discrepancies were identified and resolved by discussion. FS reviewed and approved the discussion on the five discrepancies. The process was repeated for a further ten trials (five each of the ‘SPIRIT trials’ and ‘non-SPIRIT’ trials). Six discrepancies were identified; these discrepancies were related to how to calculate the numbers recruited. It was agreed by the three authors that for the numbers recruited to the trial, we would capture the numbers present at the point of consent, but where that wasn’t available, the numbers present at the point of randomisation would be used. These numbers were located on the Consolidated Standards of Reporting Trials (CONSORT) [11] flow diagram presented in the trial publication. Where the CONSORT diagram was not present, the text was referred to, to obtain the relevant figures. Where discrepancies were identified between the text and the CONSORT flow diagram, the numbers in the CONSORT flow diagram took precedence. Due to data being imputed in many of the trials for the analysis, the figures for the ‘numbers retained’ were extracted from the component indicating direct follow-up of participants at the given time point for the primary outcome (or for further time points attributed to secondary or tertiary outcomes).

### Analysis

For each trial, the variables from the trial publication were extracted and recorded in an Excel spreadsheet. The dataset was imported into RStudio. We examined the retention rate for the primary outcome and separately for the secondary and other outcomes comparing the ‘SPIRIT trials’ and ‘non-SPIRIT’ trials. Subgroup analyses were conducted to analyse retention in academic trials and commercial trials separately. Confidence intervals were also reported. Where data was missing, the case was excluded.

## Results

In total we had 222 protocols and their corresponding trial publication, 115 of which mentioned the use of SPIRIT, ‘SPIRIT trials’, and 107 of which did not mention the use of SPIRIT, ‘non-SPIRIT’ trials.

The characteristics of the 506 trial protocols (n = 253 ‘SPIRIT’; n = 253 ‘non-SPIRIT’) are shown in Table 2. Of the 506 protocols published between 2014–2019, 56.5% (n = 286) were non-commercial and most tested non-drug interventions (68.8%, n = 348). The majority were individually randomised trials (83.6%, n = 423) and included a vast range of clinical specialities (n = 37), but the field of public health was the most common (15.8%, n = 80). Trial size also varied, ranging from 58 to 15,000 participants. The primary outcome was patient-reported in 35.6% (n = 180) of the protocols.

**Table 2:** Protocol characterises.

		Number of protocols (n = 506, %)
Year of publication	2014	40 (7.9%)
	2015	57 (11.3%)
	2016	76 (15.0%)
	2017	90 (17.8%)
	2018	92 (18.2%)
	2019	151 (29.8%)
Trial design type	Cluster RCT	83 (16.4%)
	Individually randomised RCT	423 (83.6%)
Funding type	Commercial trial^1^	48 (9.5%)
	Non-commercial trial	286 (56.5%)
	No funding	11 (2.2%)
	Unclear – no information provided	161 (31.8%)
Type of intervention	Non-drug trial	348 (68.8%)
	Drug trial	99 (19.6%)
	A mix of intervention types	18 (3.5%)
	Surgical trial	28 (5.5%)
	Medical device trial	13 (2.6%)
Patient population^2^	Vulnerable populations	151 (29.8%)
	A mix of vulnerable and non-vulnerable populations	139 (27.5%)
	Not vulnerable	207 (40.9%)
	Unclear	9 (1.8%)
Planned sample size	100 participants or less	122 (24.1%)
	101-200 participants	114 (22.5%)
	201-300 participants	64 (12.6%)
	301-400 participants	55 (10.9%)
	401–500 participants	16 (3.2%)
	>501 participants	122 (24.1%)
	Unclear from protocol	13 (2.6%)
Disease area^3^	Public health	80 (15.8%)
	Mental health	50 (9.8%)
	Obstetrics and Gynaecology	45 (8.9%)
	Musculoskeletal	41 (8.1%)
	Oncology	40 (7.9%)
	Cardiology	38 (7.5%)
	Neurology	30 (5.9%)
	Diabetes and Endocrinology	29 (5.7%)
	Respiratory	22 (4.2%)
	Sexual health and STIs	19 (3.8%)
	Paediatrics	17 (3.4%)
	Vascular diseases	16 (3.2%)
	Nephrology	12 (2.4%)
	Gastroenterology	7 (1.4%)
	Dental Health	7 (1.4%)
	Surgery and anaesthesia	6 (1.2%)
	Infectious disease	6 (1.2%)
	Intensive care	5 (1.0%)
	Otology	5 (1.0%)
	Autoimmune diseases	4 (0.8%)
	Emergency care	4 (0.8%)
	Hepatology	3 (0.6%)
	Palliative care	2 (0.4%)
	Otolaryngology	2 (0.4%)
	Dermatology	2 (0.4%)
	Haematology	2 (0.4%)
	Genetics	2 (0.4%)
	Pathology	1 (0.2%)
	Trial methods	1 (0.2%)
	Secondary care	1 (0.2%)
	Primary care	1 (0.2%)
	Pharmacy care	1 (0.2%)
	Geriatric medicine	1 (0.2%)
	Orthopaedics	1 (0.2%)
	Appendicitis	1 (0.2%)
	Renal	1 (0.2%)
	Ophthalmology	1 (0.2%)
Patient reported primary outcome	Yes	180 (35.6%)
	Partly	41 (8.1%)
	No	284 (56.1%)
	Unclear from protocol	1 (0.2%)
Number of follow-up assessments	1 follow-up assessment	74 (14.6%)
	2 follow-up assessments	133 (26.3%)
	3 follow-up assessments	105 (20.8%)
	4 follow-up assessments	67 (13.2%)
	5 follow-up assessments	19 (3.8%)
	6 follow-up assessments	68 (13.4%)
	Unclear from protocol	40 (7.9%)
Follow-up method for data collection	In-person clinic visit	177 (34.9%)
	Postal questionnaire	8 (1.6%)
	Electronic questionnaire/online assessment	30 (5.9%)
	Telephone call	15 (3.0%)
	Via patient records or databases^7^	19 (3.8%)
	Home visits/visits to site outside the clinic by researcher	16 (3.2%)
	A combination of follow-up methods	198 (39.1%)
	All data collected while participant in hospital	19 (3.8%)
	Unclear from protocol	24 (4.7%)

^1^Commercial trials were defined as a trial that received any type of funding or donation from a private for-profit company/organization i.e., any donation or part-donation received from a pharmaceutical company resulted in that trial being deemed as commercial.

^2^Vulnerable populations were defined via local ethics committee and ICH-GCP definition [12]. Disease area was categorized based on clinical speciality, for example, ICU admittance due to infectious disease contraction was categorized as infectious disease rather than intensive care. Only ICU admittance for non-specific or unlisted reasons would be categorized as intensive care.

### SPIRIT item 18b reporting in the trial publication

Table 3 reports the key findings concerning the use of SPIRIT items 18b (i), (ii) and (iii). Of the 222 trial publications (n = 115 ‘SPIRIT’; n = 107 ‘non-SPIRIT’) no publication included all aspects of item 18b (18b (i), (ii) and (iii) – “plans to promote participant retention and complete follow-up, including list of any outcome data to be collected for participants who discontinue or deviate from intervention protocols”(10:3). Thirty-one-point-three percent (36/115) of ‘SPIRIT trials’ reported on item 18b(i), “plans to promote participant retention” in comparison to 1.9% (2/107) in the ‘non-SPIRIT trials’. Three-point-five percent (4/115) of ‘SPIRIT trials’ reported on item 18b(ii), “plans to complete follow-up including list of any outcome data to be collected for participants who discontinue from intervention protocols”, in comparison to 0% in the ‘non-SPIRIT trials’. Finally, no trials in either the ‘SPIRIT trials’ or ‘non-SPIRIT trials’ reported on item 18b(iii), “plans to complete follow-up including list of any outcome data to be collected for participants who deviate from intervention protocols”.

**Table 3:** Key SPIRIT characteristics observed in trial publications.

	Number of trial publications (n = 222)
Mentioned the use of the SPIRIT in the journal article	
SPIRIT trials	5 (4.3%)
Non-SPIRIT trials	0 (0%)
Reported item 18b(i) “Plans to promote participant retention”	
SPIRIT trials	36 (31.3%)
Non-SPIRIT trials	2 (1.9%)
Reported item 18b(ii) “Plans to complete follow-up including list of ant outcome data to be collected for participants who discontinue from intervention protocols”	
SPIRIT trials	4 (3.5%)
Non-SPIRIT trials	0 (0%)
Reported item 18b(iii) “Plans to complete follow-up including list of ant outcome data to be collected for participants who deviate from intervention protocols”	
SPIRIT trials	0 (0%)
Non-SPIRIT trials	0 (0%)
Reported all aspects of item 18b “plans to promote participant retention and complete follow-up, including list of any outcome data to be collected for participants who discontinue or deviate from intervention protocols”	
SPIRIT trials	0 (0%)
Non-SPIRIT trials	0 (0%)

### Trial retention

The logistic regression results show that the use of SPIRIT guidelines, item 18b, in the protocol did not improve participant retention for all reported trial outcomes as the confidence interval included the value of no association. When considered separately, findings for retention to the primary outcome were similar. For commercial trials, and academic trials, the results were no different as can be seen from the confidence intervals in Table 4.

**Table 4:** Logistic regression.

	Overall retention	Retention for primary outcome	Retention in commercial trials	Retention in academic trials
Coefficient estimate	0.3858	1.0967	1.606	0.2342
Std. error	0.37022	0.5919	1.0816	0.4076
Z-value	1.042	1.853	1.073	0.575
Odds Ratio	1.47	2.99	2.32	1.16
95% CI Lower	0.71	0.92	0.7	0.5
95% CI Upper	3.03	9.66	7.7	2.7

## Discussion

Our analyses suggest that the reported use of the SPIRIT guidelines does not improve participant retention rates in trials, but this hides the reality of what is happening in practice. Despite 115 ‘SPIRIT trials’ (protocols that mentioned the use of SPIRIT guidelines) [10] when developing the trial protocol, often there was no evidence of SPIRIT item 18b in the resulting trial publication. Among the identified trial publications corresponding to protocols in the SPIRIT group, 31.3% (36/115) reported item 18b(i), 1.7% (2/115) reported item 18b(ii) and none of the journal articles reported item 18b(iii). This could be because the trial team did not consider participant retention during protocol development and therefore did not include a “plan to promote participant retention” (SPIRIT item 18b(i)) despite the guidance outlined in the SPIRIT guidelines, or that SPIRIT item 18b was considered but not reported by the trial team in the resulting trial publication. While SPIRIT is endorsed as an international standard for clinical trial protocols, its use is not mandatory, so thorough adherence to the guidelines may not be prioritised. Due to the lack of reporting of SPIRIT item 18b we are not able to determine if trial teams have in-fact used strategies to promote retention of participants in the trials. Neither can we thus determine if proactive strategies (plans outlined in the protocol that aim to actively promote participant retention in the trial) or reactive strategies (plans outlined by the trial team to complete outcome data collection and to complete follow-up of participants who have withdrawn/discontinued or deviated from the intervention protocols) were used, an issue we discussed in our scoping review previously [9]. This contributes to the issue of incomplete reporting and causes a lack of transparency in trial conduct [10,13–15]. This lack of reporting ultimately limits trial methodology researchers when attempting to carry out replications and evaluations of retention strategies which are very much needed due to the lack of evidence to support the effectiveness of these activities [1].

Further reporting issues were also identified in this paper. We found a disparity in the reporting of recruitment and retention figures, where seemingly, researchers interpret the ‘point of enrollment’ differently across several trials. We also found several disparities between the consort diagram and the text in the trial publication. Therefore, improved reporting is crucial for transparency and to facilitate readers in obtaining an unambiguous comprehension of what occurred throughout the trial. Ensuring consistency in reporting standards across trials will enhance the reliability and reproducibility of research findings, ultimately strengthening the evidence base for clinical practice.

It might also be the case that the SPIRIT guidelines for trial retention (item 18b) may lack specificity. Item 18b states “plans to promote participant retention and complete follow-up, including list of any outcome data to be collected for participants who discontinue or deviate from intervention protocols”(10:3), however, it’s possible that more inexperienced trial teams are unsure what constitutes a retention strategy. For example, it might be standard practice in their clinical trial unit to phone trial participants to remind them of their upcoming visit. If it is an activity that is routine, perhaps they do not view it as a ‘retention strategy’. Both SPIRIT 2013 [10] and CONSORT (consolidated standards of Reporting Trials) 2010 [16] have been updated simultaneously with the aim of aligning reporting in both checklists and to provide unified guidance for reporting trial design, conduct and analysis from protocol development to final publication. The initial thought given the overlap between SPIRIT and CONSORT, particularly in methodological aspects of trial design, was that alignment would enhance practicality, facilitate implementation and improve efficiency [17]. The new guidelines have been published and while both SPIRIT 2025 [18] and CONSORT 2025 [19] have increased in length, little has changed to harmonise the planning (SPIRIT) and reporting (CONSORT) of recruitment strategies. The language has remained identical between item SPIRIT 2013 and SPIRIT 2025. SPIRIT 2025 Item 25b (18:38) “Plans to promote participant retention and complete follow-up, including list of any outcome data to be collected for participants who discontinue or deviate from intervention protocols” states that planning is desirable, rather than essential, but acknowledges the effect on the trial analysis and interpretation of results “…desirable to plan for how retention will be promoted to prevent missing data and avoid the associated complexities in both study analysis (item 27c) and interpretation”. CONSORT 2025 has no reporting requirement for retention strategies used during the trial. Consort Item 22b requires information on losses and exclusions “For each group, losses and exclusions after randomisation, together with reasons” but does not expand this to seek information on efforts made to encourage trial participants to stay in the trial. It is perhaps a missed opportunity.

While a lack of SPIRIT guideline usage and/or reporting is a possible explanation of the resulting non-significance in this research, it is also important to consider the possibility that the retention strategies used in the trials were not effective enough to overcome the challenges associated with participant retention. There were a variety of different strategies mentioned across both the SPIRIT group and the non-SPIRIT group, for example, offering the control group active treatment following trial culmination [20], offering monetary incentives to participants at the last patient visit [21] or reminding participants of appointments via phone call or text message [22]. These commonly used strategies may not be the most effective. In fact, evidence from the Cochrane systematic review of retention strategies in trials shows that there is no high-certainty evidence to support any existing documented retention strategy [1]. This is quite stark.

Finally, we did not see any instances of all three items being completed (18b(i), (ii), and (iii)). It would be interesting to assess if all these elements of SPIRIT item 18b were completed, which would indicate forward planning for retention to the trial, what the impact on the overall retention to the trial would be. We cannot answer that now, but we call on all trial teams to plan strategies to improve retention to their trial early and document these plans. This will facilitate replication and evaluation in the future.

### Strengths and limitations

Including protocols from 37 different clinical specialties allowed us to represent a diverse patient population with varying demographic characteristics and medical conditions. This strengthens the external validity of the research and thus the generalisability of the findings. A limitation of our study is that we included study protocols from up to 2014–2019 in our research. Given that this research was carried out in early 2023, 284 of the 506 trial protocols had no trial publications associated with them as some of the trials were still recruiting or ongoing, and some had not yet published the findings. This diminished our sample size to an extent, and so it would be interesting to assess the impact of the use of SPIRIT item 18b again when all 506 have results published. Similarly, the lack of trial publications reduced our planned sample size for analysis, and this may explain the wide confidence intervals and non-significant findings.

### Implications for practice

These findings should be interpreted considering the Cochrane systematic review of retention strategies which reports no high-certainty evidence as determined by GRADE assessment (a systematic approach to rating the certainty of evidence in systematic reviews and evidence syntheses). Thus, expecting an impact on trial retention here is perhaps premature. The key message from this paper is that there is a lack of planning for retention in trials. It should happen early in the trial, and the recording of those plans via SPIRIT, to facilitate replication and evaluation is of vital importance. Only then, with this evidence, can we truly identify if using SPIRIT item 18b (25b in the new SPIRIT 2025) when writing the trial protocol, will impact the retention rate in the trial. Reporting of any strategies used is also vital in the trial report.

## Conclusions

High levels of attrition in randomised trials can result in both poor internal and external validity [1,3,7,8,23,24], which can be detrimental to trial outcomes. While we cannot conclude from this research that SPIRIT item 18b has the capability of improving participant retention in trials, we believe this is attributable to the lack of evidence on the existing strategies being used, rather than a reflection on SPIRIT itself. We strongly encourage all trial teams to use the SPIRIT guidelines [10] as this contributes to the overall quality, completeness, transparency, and consistency of trial reporting [9,17,24,25]. Raising awareness of the need for early planning for participant retention along with the increased reporting of item 18b may well help to address the considerable challenge, that is participant retention [4].
